# Giant enhancement of superconducting critical temperature in substitutional alloy (La,Ce)H_9_

**DOI:** 10.1038/s41467-022-33743-6

**Published:** 2022-10-10

**Authors:** Jingkai Bi, Yuki Nakamoto, Peiyu Zhang, Katsuya Shimizu, Bo Zou, Hanyu Liu, Mi Zhou, Guangtao Liu, Hongbo Wang, Yanming Ma

**Affiliations:** 1 State Key Laboratory of Superhard Materials and International Center of Computational Method & Software, College of Physics, Jilin University, 130012 Changchun, China; 2 Center for Quantum Science and Technology under Extreme Conditions, Osaka University, Toyonaka, Osaka 560-8531 Japan; 3 International Center of Future Science, Jilin University, 130012 Changchun, China

**Keywords:** Superconducting properties and materials, Superconducting properties and materials

## Abstract

A sharp focus of current research on superconducting superhydrides is to raise their critical temperature *T*_c_ at moderate pressures. Here, we report a discovery of giant enhancement of *T*_c_ in CeH_9_ obtained via random substitution of half Ce by La, leading to equal-atomic (La,Ce)H_9_ alloy stabilized by maximum configurational entropy, containing the LaH_9_ unit that is unstable in pure compound form. The synthesized (La,Ce)H_9_ alloy exhibits *T*_c_ of 148–178 K in the pressure range of 97–172 GPa, representing up to 80% enhancement of *T*_c_ compared to pure CeH_9_ and showcasing the highest *T*_c_ at sub-megabar pressure among the known superhydrides. This work demonstrates substitutional alloying as a highly effective enabling tool for substantially enhancing *T*_c_ via atypical compositional modulation inside suitably selected host crystal. This optimal substitutional alloying approach opens a promising avenue for synthesis of high-entropy multinary superhydrides that may exhibit further increased *T*_c_ at even lower pressures.

## Abstract (web summary)

Superconductivity was recently discovered in the clathrate hydride CeH_9_ with superconducting temperature (*T*_c_) of 57 K at pressures below 1 megabar. Here, the authors show that *T*_c_ can be increased to 148 K in the substitutional alloy (La,Ce)H_9_, while maintaining a pressure below 1 megabar.

## Introduction

Ever since Heike Kamerlingh Onnes made the astonishing discovery^1^ that the electrical resistance of mercury disappeared as temperature dropped to a few Kelvins via liquid-helium cooling, this exotic quantum phenomenon, known as superconductivity, has attracted tremendous interest in fundamental scientific inquiry and spurred intense efforts for applications ranging from detection of faint magnetic signals to generation of intensive magnetic fields. The long-sought overarching goal is to find materials with sufficiently high superconducting critical temperatures (*T*_c_s) to facilitate experimental exploration and practical implementation, ultimately allowing ambient-environment applications. Over the years, the search for room-temperature superconductors has been among the most luring and challenging topics in condensed matter physics; notable progress includes materials with *T*_c_ in liquid-hydrogen and liquid-nitrogen temperature ranges achieved at ambient pressure^2,3^, making it more easily manageable to utilize superconductors in advanced devices. More recently, superconductivity with *T*_c_ approaching or even exceeding the room-temperature has been predicted in a class of hydrogen-rich compounds, termed superhydrides, under extreme compression. Theoretical prediction followed by experimental synthesis led to the discovery of superconductors with *T*_c_ of 203 K in covalent SH_3_^4–6^ and 250–260 K in ionic LaH_10_^7–10^, obtained at very high pressures between 150–200 GPa. A *T*_c_ of 288 K was reportedly achieved at extremely high pressures around 270 GPa in a C-S-H system with unknown composition and structure^11^, but it has caused intense debate^12–14^ and still awaits confirmation.

Meanwhile, inspired by the prediction of high-temperature superconductive CaH_6_^15^, extensive ensuing studies have established a large family of clathrate superhydrides, including CaH_6_^16,17^, YH_6_^18^, YH_9_^19,20^, CeH_9_^21–23^, CeH_10_^21^, and LaH_10_^9,10^, which comprise an array of distinct hydrogen cages anchored at the center by a variety of metal atoms. The diverse structural and compositional forms of these binary compounds offer a platform for exploring material parameters to optimize the energetic and superconducting states. Systematic studies indicate that atomic radius, electronegativity, and valence electron of metal atom play important roles in tuning the most concerned properties of superhydrides, i.e., superconductivity and stability^24^. Among these ionic superhydrides, LaH_10_ faces an inescapable problem of its harsh synthesis pressure (>150 GPa), despite its very high *T*_c_^9,10^; whereas CeH_9_ or CeH_10_ with relatively low *T*_c_ of 95–115 K can be synthesized around megabar pressure^21^. Improving the superconducting properties of these superhydrides with moderate synthesis and stability pressures presents pressing challenges in this active research field.

Binary superhydrides are highly restricted in their configurational space with limited structural and compositional variations. To expand the material horizon, related research recently shifted focus to ternary systems with much higher degrees of freedom, which offer a larger number and richer variety of structural prototypes for superconducting superhydride screening^25^. The potential of this approach is demonstrated in a theoretical work^26^ showing that Li doping introduces an extra electron into the molecular-like hydrogen in MgH_16_ to generate atomic-like hydrogen, and the formed ternary Li_2_MgH_16_ has a *T*_c_ of 473 K at 250 GPa. In addition, theoretical studies have also designed a series of ternary superhydrides^25,27^, such as XYH_8_^28–30^, Ca-Y-H^31^, and Ca-Mg-H^32^ that are expected to possess desirable high-*T*_c_ features or prospects. A sticking point, however, is to find ternary superhydrides that host high-temperature superconductivity at moderate, near or below megabar, pressures.

Rare-earth (RE) metals have similar electronegativities, electronic configurations, and atomic radii, so their disordered solid solution alloys are easy to form^33,34^. This offers a viable avenue to use suitably selected binary compounds as the base template to construct ternary alloy superhydrides that share the same crystal structure. It has been shown that LaH_6_ and YH_10_ units, which are experimentally unreachable in the binary systems, do appear in La-Y alloy hydrides at pressures of 170–196 GPa^35^, although superconductivity is not improved. It is of great interest to explore the cubic La-H^9,10,36^ and hexagonal close packed (*hcp*) Ce-H^21–23^ systems to find a desirable ternary platform for experimental realization of high-temperature superconductivity in materials that can be stabilized at relatively low pressures.

In this work, we experimentally investigated the crystal structure, superconductivity, and stability pressure range of a La-Ce alloy superhydride. Substitutional (La,Ce)H_9_ with essentially equal metal-atom occupancy was obtained using the starting materials of equiatomic La-Ce alloy and ammonia borane (NH_3_BH_3_) under the synthesis conditions of about 110 GPa and 2100 K. The synthesized ternary alloy was maintained to at least 90 GPa during decompression. Compared with binary CeH_9_, the *T*_c_ is dramatically increased by up to 80 K in ternary (La,Ce)H_9_, which contains an unprecedented LaH_9_ unit that is unstable in pure compound form but stabilized in the base binary CeH_9_ structural framework. This atypical compositional structure unit, together with the reduction of possible Abrikosov-Gor’kov suppression-like effect^37^ of Ce by replacing with La, have a critical impact on the enhancement of *T*_c_ of the ternary (La,Ce)H_9_ alloy. The present findings demonstrate that substitutional alloying is effective in tuning and improving high-temperature superconductivity in superhydrides by stabilizing new and unusual structural and compositional configurations that are conducive to greatly enhanced *T*_c_, and this approach may be extended to constructing additional ternary and higher multinary alloys to usher in more breakthrough discoveries in the quest of finding optimal clathrate superhydrides through a thoughtful choice of substitutional alloying metal-element combinations.

## Results

### Synthesis of ternary (La,Ce)H_9_ alloy superhydride

**Fig. 1:**
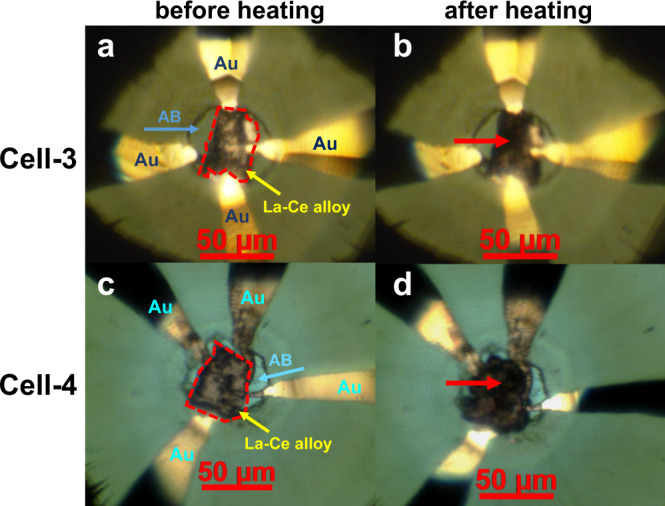
Optical micrographs of the sample chambers containing NH_3_BH_3_ (AB) and Au electrodes in Cell-3 and Cell-4 before and after laser heating. **a** and **b** are photos of Cell-3 before and after laser heating, respectively. **c** and **d** are photos of Cell-4 before and after laser heating, respectively. The edges of La-Ce alloys are marked with red dotted lines, and the red arrows point to the parts with apparent changes after the heating.

We took a flake of La-Ce alloy sample from a homogeneous region of the inside part of the specimen and loaded it into a diamond anvil cell (DAC) with NH_3_BH_3_ as the pressure transmitting medium and hydrogen source. A total of seven samples were compressed to 110–125 GPa at room-temperature and then heated to 2100–2300 K with pulsed radiation from an yttrium-aluminum-garnet laser (Supplementary Table 1). The color of the sample changed significantly after laser spot irradiation (Fig. 1), indicating that the expected chemical reaction occurred. After keeping the synthesized samples under high pressure while quenching to room-temperature, we performed subsequent structure and superconductivity characterizations.

### Crystal structure of synthesized (La,Ce)H_9_

**Fig. 2:**
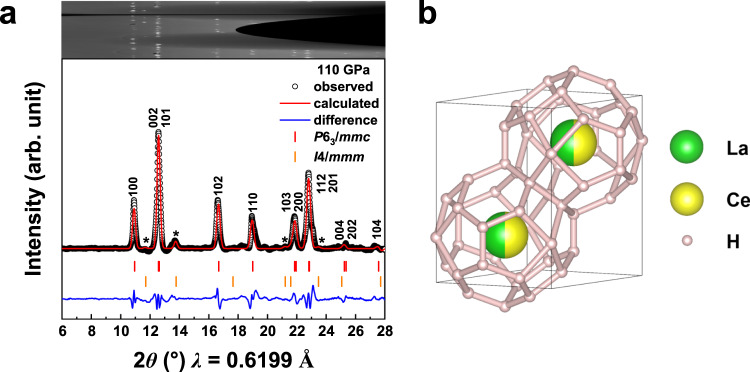
Structural data for (La,Ce)H_9_ synthesized from La-Ce alloy and NH_3_BH_3_. **a** Synchrotron X-ray diffraction pattern of the La-Ce alloy hydrides obtained following laser heating and the Rietveld refinement of the *P*6_3_/*mmc*-(La,Ce)H_9_ structure at 110 GPa. The cake view of raw XRD pattern is shown in the upper inset. The high-intensity points caused by single-crystal-like diffraction (e.g., the points at ~11.6° in the inset) are masked, and the continuous background is removed before performing the integration and Rietveld analysis. The Rietveld fit parameters are *R*_wp_ = 0.46% and *R*_p_ = 0.80%. The weak peaks marked with asterisks are from the *I*4/*mmm*-tetrahydride. **b** Schematic diagram of the crystal structure of *P*6_3_/*mmc*-(La,Ce)H_9_.

**Fig. 3:**
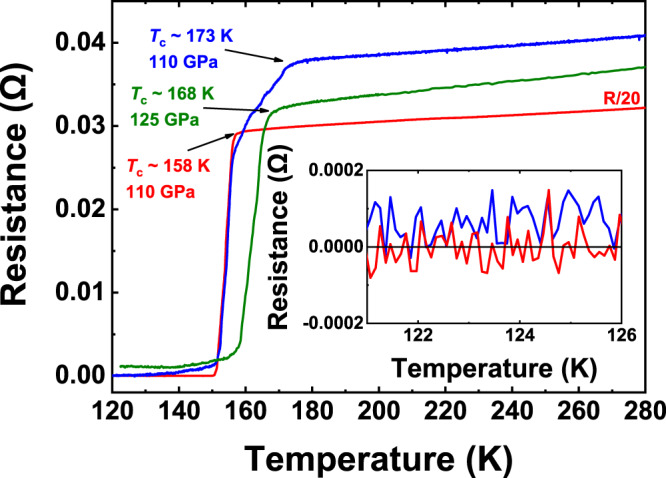
Superconducting transitions determined by electrical resistivity measurements in typical cells. Resistance measurements of synthesized (La,Ce)H_9_. Red curve: sample at 110 GPa with *T*_c_ ~ 158 K. Green curve: sample at 125 GPa with *T*_c_ ~ 168 K. Blue curve: sample at 110 GPa with *T*_c_ ~ 173 K. The near-zero resistance is shown on a smaller scale in the inset.

**Fig. 4:**
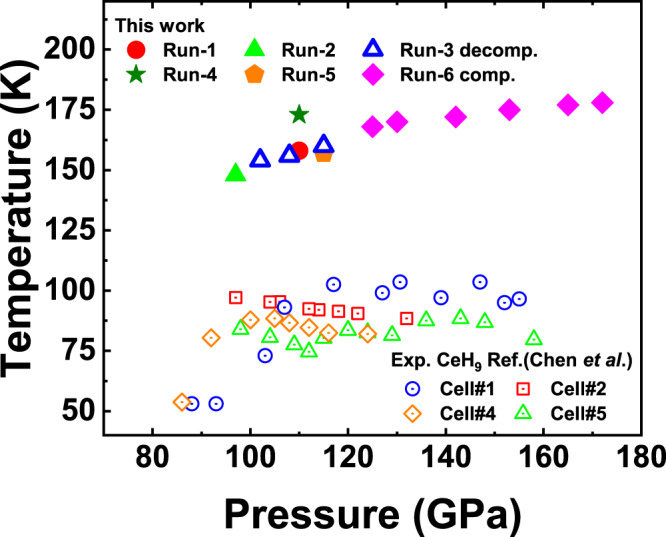
*T*_c_ versus pressure for *P*6_3_/*mmc*-(La,Ce)H_9_. The variation of superconducting critical temperature *T*_c_ with changing pressure; the results from six different experiments are marked in different colors. Comparison data on CeH_9_ were taken from work by Chen et al. reported in ref. 21.

To determine the crystal structure of synthesized La-Ce superhydride, we conducted in-situ X-ray diffraction (XRD) experiments in synchrotron radiation sources. The representative XRD pattern of the product in Cell-4 at 110 GPa is shown in Fig. 2a. The observed peaks can be indexed by an *hcp* (*P*6_3_/*mmc*) lattice with cell parameters of *a* = 3.762(6) Å and *c* = 5.675(5) Å (Supplementary Table 2). The weak peaks marked with asterisks are from the tetragonal tetrahydride reported in previous studies^21,22^, which is a common occurrence caused by temperature or pressure gradients during high-temperature and high-pressure synthesis. Notably, in this measured *hcp* lattice, there is only one Wyckoff 2d metal position, but it has to accommodate two different elements of La and Ce, each of which occupies the metal position at a probability of ~50% since the molar ratio of La: Ce is at ~1:1. As a result, there is a formation of a substitutional *hcp* La/Ce alloy in the metal lattice. Since La and Ce share highly similar X-ray scattering factors, it is impossible to determine the La/Ce ratio through the Rietveld refinement. Instead, we determine the ratio by a true observation. We note that in the XRD data, besides the peaks for the synthesized product, there is absence of any peaks that correspond to those for elemental La and Ce phases. This suggests that the precursor of La-Ce alloy with a molar ratio of La: Ce at ~1:1 (Supplementary Tables 3–5 and Figs. 1–4), fully reacts with hydrogen for the formation of hydrides. As a result, La/Ce ratio in the formed hydride can be concluded as 50% to 50%. A similar *hcp* structure mixed with tetrahydride was successfully reproduced at 115 GPa in another independent experimental run (Supplementary Figs. 5 and 8), corroborating the reliability of this structure.

Although the occupancy details of the hydrogen atoms cannot be determined experimentally due to the weak X-ray scattering cross section, the measured unit cell volume (*V*_lattice_) can be used to estimate the hydrogen concentration of the superhydride. While the volumes (*V*_La_, *V*_Ce_, and *V*_H_) of La, Ce, and H atoms are not known, they have to be estimated from their elemental phases. Specifically, at 110 GPa, phase structures of distorted-*fcc* for La^38^, of *bct* for Ce^39^, and of *hcp* for H_2_^40^ are adopted where *V*_La_, *V*_Ce_, and *V*_H_ are calculated to be ~16.04, ~14.59, and ~2.30 Å^3^/atom, respectively. As a result, the hydrogen content (*n*) of the synthesized La-Ce alloy hydrides was determined as ~8.5 using the formula of *n* = (*V*_lattice_ – *V*_La _– *V*_Ce_)/*V*_H_/2, which has a small deviation with the ideal value of 9. Numerical error in this calculation is inevitable, and it comes from the direct use of elemental phases for the evaluation of volumes. It is expected that once a compound is formed the volume of constituted elements will somewhat change compared with their elemental phases. Furthermore, the experimental equation of state (EOS) during decompression is highly consistent with the simulated EOS (Supplementary Fig. 6), where most of the experimental points lie between the lines of *hcp* CeH_9_ and hypothetical *hcp* LaH_9_. Combined with the composition of the original alloy sample, our diffraction experiments show that ~50% of the Ce atoms in the recently discovered structure of *P*6_3_/*mmc*-CeH_9_ are replaced by La, forming a unique ternary alloy superhydride *P*6_3_/*mmc*-(La,Ce)H_9_ (Fig. 2b).

### Superconductivity in (La,Ce)H_9_

To probe superconductivity in the synthesized alloy superhydride, we performed electrical transport measurements. Representative measured electrical resistance data as a function of temperature are shown in Fig. 3, which clearly shows the superconducting transition, as indicated by the sharp drops in resistance occurring at 158 K, 168 K, and 173 K at about 110 GPa, 125 GPa, and 110 GPa, respectively. In these experiments, zero resistance states were observed in Cell-1 and Cell-4 (Fig. 3 inset). To determine the highest value of *T*_c_, we proceeded to regulate the pressure dependence on *T*_c_, as shown in Fig. 4. With the increase of pressure, *T*_c_ increases first and then tends to be flat. The highest *T*_c_ of 178 K in this work was observed at 172 GPa (Supplementary Figs. 9–14). Our Rietveld refinement of the synthesized products indicates that the major (~96.2 At%) phase is *P*6_3_/*mmc*-(La,Ce)H_9_ with a tiny amount of impurity (~3.8 At%) being as *I*4/*mmm*-(La,Ce)H_4_ (Fig. 2a). In our four-probe van der Pauw experiment for the superconductivity measurement, the chance is extremely low if the four separate electrodes simultaneously touch the minor phase of the sample. Notably, our experimental superconductivity is quite reproducible at least for six different runs of experiments. This suggests that the experimentally observed superconductivity is unlikely originated from the minor phase. Moreover, it should also be pointed out that the minor phase of *I*4/*mmm*-(La,Ce)H_4_ would not hold such high superconductivity at *T*_c_ = 148–178 K observed in this study. For a good comparison, its isostructural phase in a lighter hydride of YH_4_ shows much lower superconductivity at 76–88 K^41–43^. It is expected that the heavier hydride of (La,Ce)H_4_ should possess even lower *T*_c_ values than that in YH_4_. Therefore, the measured superconductivity is associated with major (La,Ce)H_9_ rather than minor (La,Ce)H_4_. With decreasing pressure, the superconducting transition tends to disappear at about 90 GPa (Supplementary Fig. 11), indicating a probable decomposition of the superconducting phase.

**Fig. 5:**
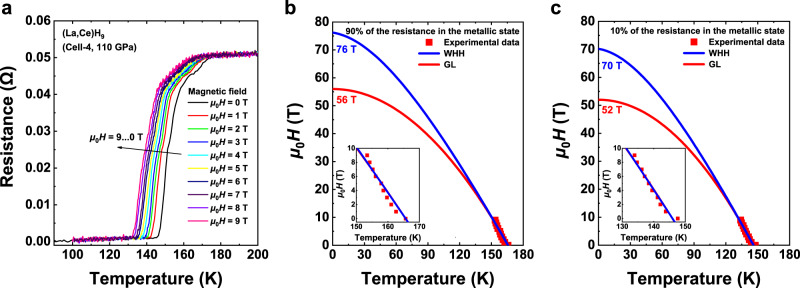
Electrical measurements in the external magnetic field of the synthesized (La,Ce)H_9_. **a** Temperature dependence of the electrical resistance under applied magnetic fields of *μ*_0_*H* = 0–9 T at 110 GPa. **b** and **c** Upper critical field *μ*_0_*H*_c2_ versus temperature following the criterion of 90% and 10% of the resistance in the metallic state at 110 GPa, fitted with the GL^44^ and WHH^45^ models. Inset: the dependence of the *T*_c_ under the applied magnetic field.

Owing to the rather small size of the samples, it is still a challenging task to measure the weak signals of the magnetic flux expulsion (i.e., Meissner) effect under high pressures^10,20^. However, due to the Pauli paramagnetic effect of electron spin polarization and the diamagnetic effect of orbital motion, an applied external field can disrupt the Cooper pairs, thereby reducing the value of *T*_c_. As shown in Fig. 5a, the resistance drop gradually shifts to lower temperatures as the magnetic field increases in the range of 0–9 T at 110 GPa. Combined with the observed zero resistance, it can rule out the possibility that the abrupt drop of resistance on cooling arises from structural transitions. The upper critical field as a function of temperature, defined as 90% (10%) of the resistance, is shown in the inset of Fig. 5b, c. At *μ*_0_*H* = 9 T, the application of the magnetic field reduces *T*_c_ by about 12 K (14 K). The extrapolated values of the upper critical field *μ*_0_*H*_c2_(*T*) and the coherence lengths towards *T* = 0 K are 56 T (52 T) and 24 Å (25 Å) and 76 T (70 T) and 21 Å (22 Å), respectively, by Ginzburg-Landau (GL)^44^ and Werthamer-Helfand-Hohenberg (WHH)^45^ model fits. The obtained *μ*_0_*H*_c2_ data and coherence lengths following the 10% criterion of the metallic state resistance are similar to those derived from the use of 90% resistance. The magnitude of the short coherence lengths and high upper critical fields indicate that ternary (La,Ce)H_9_ is a typical type-II superconductor. We note that the similar superconductivity of ternary La-Ce-H system was observed by two different groups^46,47^ during the preparation of our manuscript.

## Discussion

The clathrate REH_9_ phase with H_29_ cage was predicted^8^ to be a high-temperature superconductor in 2017, and this structural prototype has since been experimentally verified by a series of RE metal superhydrides^48–51^. Of particular interest among these compounds is CeH_9_ that was synthesized at easily achievable megabar pressure and exhibited an experimental *T*_c_ of ~95 K^21^. Compared to the higher-*T*_c_ LaH_10_^9,10,36^, the low *T*_c_ of CeH_9_ stems from its low logarithmic average phonon frequency, which may be related to the 4*f* electron in Ce at high pressure^24^. Furthermore, no superconductivity or only low-temperature superconductivity was observed in other similar RE metal superhydrides, where strongly correlated interactions of 4*f* electrons dramatically suppress the superconductivity^24,48–52^. It has been reported that the superconductivity of LaH_10_ was dramatically suppressed by doped Nd^53^ (each atomic percent of Nd causes a decrease in *T*_c_ of 10–11 K), which can be reasonably understood by the well-known impure superconductor theory proposed by Abrikosov and Gor’kov^37^. If half of the weakly magnetic Ce in CeH_9_ is replaced by non-magnetic La, Abrikosov-Gor’kov suppression-like effect of Ce should be significantly weakened, resulting in the giant lift of *T*_c_ in (La,Ce)H_9_ over that in CeH_9_. This analysis points to rational improvement of superconductivity in CeH_9_ via substitution of Ce by La that hosts no 4*f* electron but otherwise shares most chemical characters.

In addition, the pressure dependence of *T*_c_ varies slightly in different experiments, which may originate from subtle differences in molar ratios of the La-Ce alloy polyhydride samples or different degrees of anisotropic stress that leads to variable deformation of the lattice in different experiments^54^. Moreover, the observed *c*/*a* ratio (1.511) of the synthesized *hcp* (La,Ce)H_9_ at 110 GPa is smaller than the ideal value of 1.633. This indicates that the structure is not isotropic, which may play somewhat role in regulating superconductivity.

Although *hcp* LaH_9_ is calculated to be thermodynamically unstable^8^ and unlikely to be synthesized among binary system at the corresponding pressure of ~100 GPa, the LaH_9_ structural unit appears in synthesized ternary La-Ce alloy superhydride via La-substitution modification of the native CeH_9_ units. This result can be elucidated by the Hume-Rothery rule^55^, because the ratio of La/Ce atomic radii (~1.032 at 110 GPa)^38,39^ is well within the rule’s stated 15% range, and La and Ce have similar electronegativity values^33^, thus satisfying the conditions for the formation of a disordered solid solution compound. Moreover, laser heating helps to overcome the kinetic reaction barrier and stabilize substitutional (La,Ce)H_9_ via the contribution of the configurational entropy to the total free energy, overcoming the contribution of mixing enthalpy and the energy increment of LaH_9_ relative to LaH_10_ at a sufficiently high temperature^34^. In particular, the configurational entropy reaches the maximum in an equal-atomic distribution and plays a significant role in stabilizing ternary (La,Ce)H_9_. This effect should be more pronounced in higher multinary superhydrides, such as equiatomic medium- or high-entropy alloy superhydrides. Further theoretical examination and experimental verification are needed.

In conclusion, we discovered a giant *T*_c_ enhancement in equal-atomic substitutional ternary alloy (La,Ce)H_9_ that exhibits high *T*_c_ values of 148–178 K in the pressure range of 97–172 GPa examined in this work. These *T*_c_ values are significantly higher, by up to 80 K, compared to the results found in binary CeH_9_, which represents a giant, 80% enhancement. This ternary alloy superhydride integrates the native CeH_9_ structural units and the atypical LaH_9_ units that are unstable in pure compound form but stabilized by optimal configurational entropy under the laser-heated DAC compression synthesis conditions. These results show that substitutional alloying is an effective and promising approach to tune and improve superconducting superhydrides, leading to the long-sought scenario of high-temperature superconductivity at relatively lower pressures. The present findings also provide insights for expanding the scope of ongoing studies in search of room-temperature superconductors among diverse compounds via construction of multinary alloys via suitable metal-atom substitution.

## Methods

### Alloy preparation

La-Ce alloy (commercial product, 1:1 in molar ratio) was prepared by levitation melting technology, where mixed elemental La and Ce with purity of 99.7% were heated to completely melt in an Ar atmosphere at a pressure of 60 KPa for 1–2 min and then were annealed for about 40 min. To ensure the uniformity of the composition, the specimen ingot needed to be turned over and the above process was repeated more than twice. The 1:1 molar ratio and homogeneity of La-Ce alloy were further confirmed by our own experiments using inductively coupled plasma atomic emission spectroscopy (Thermo Fisher iCAP PRO) and scanning electron microscope (JEM-2000FS, Regulus 8100, and ZEISS Gemini 300) equipped for the energy dispersive X-ray spectroscopy.

### Synthesis in diamond anvil cell

We synthesized the superhydrides via a reaction of La-Ce alloy and NH_3_BH_3_ (Sigma-Aldrich, 97%) in diamond anvil cells (DACs). The diamond anvils with culets of 60–100 µm in diameter were beveled at 8.5° to about 250 µm in diameter. A composite gasket consisting of a rhenium outer annulus and an aluminum oxide (Al_2_O_3_) epoxy mixture insert was employed to contain the sample while isolating the electrical leads in the electrical measurements. La-Ce alloy foil with a thickness of 2–3 µm was sandwiched between NH_3_BH_3_. Sample preparation and loading were done in an inert Ar atmosphere with residual O_2_ and H_2_O contents of <0.01 ppm to guarantee that the sample was properly isolated from the surrounding air atmosphere. The samples were compressed to target pressures at room-temperature. The pressure values in cells were determined from room-temperature first-order diamond Raman edge calibrated by Akahama^56,57^. One-side laser-heating experiments were performed using a pulsed YAG laser (1064 nm) with spots of ~10 μm in diameter. The temperature was determined using the emission spectrum of the black body radiation within Planck’s radiation law.

### Structure characterization

X-ray diffraction (XRD) patterns were obtained at BL15U1 (*λ* = 0.6199 Å) of Shanghai Synchrotron Radiation Facility and BL10XU (*λ* = 0.4131 Å) of at the SPring-8 facility^58^ with focused monochromatic X-ray beams (5 × 12 and 3 × 2 µm^2^). A Mar165 CCD detector and an imaging plate detector (RAXIS-IV; Rigaku) were used to collect the angle-dispersive XRD data. The sample to detector distance and other geometric parameters were calibrated using a CeO_2_ standard. The software package Dioptas was used to integrate powder diffraction rings and convert the 2-dimensional data to 1-dimensional profiles^59^. The full profile analysis of the diffraction patterns and the Rietveld refinements were done using GSAS and EXPGUI packages^60^.

### Electrical transport measurement

The resistance was all measured via the four-probe van der Pauw method where four Au electrodes were placed on the NH_3_BH_3_ plate with currents of 1–100 µA. The temperature dependence of the electrical resistance was measured upon cooling and warming cycles with a slow temperature ratio (0.2–0.3 K min^−1^). The data was taken upon warming, as it yielded a more accurate temperature reading. Symmetric Cu-Be alloy DACs were used for electronic transport measurements under external magnetic fields up to 9 T.

## Supplementary information

Supplementary Information

## Data Availability

The authors declare that the main data supporting the findings of this study are contained within the paper and its associated Supplementary Information. All other relevant data are available from the corresponding author upon reasonable request.
