# Supplementary material for: Giant enhancement of superconducting critical temperature in substitutional alloy (La,Ce)H9
Source: Nat Commun. 2022 Oct 10;13:5952. doi: 10.1038/s41467-022-33743-6 (PMC9551097; doi:10.1038/s41467-022-33743-6)
Supplement: Supplementary file 1 — Supplementary Information [file 41467_2022_33743_MOESM1_ESM.pdf]

Supplementary Information

for

"Giant enhancement of superconducting critical temperature in substitutional alloy $(La,Ce)H_9$"

Jingkai Bi[1], Yuki Nakamoto[2], Peiyu Zhang[1], Katsuya Shimizu[2], Bo Zou[1], Hanyu Liu[1], Mi Zhou[1], Guangtao Liu[1, *], Hongbo Wang[1, *], and Yanming Ma[1, 3, *]

[1]*State Key Laboratory of Superhard Materials and International Center of Computational Method & Software, College of Physics, Jilin University, Changchun 130012, China*

[2]*Center for Quantum Science and Technology under Extreme Conditions, Osaka University, Toyonaka, Osaka 560-8531, Japan*

[3]*International Center of Future Science, Jilin University, Changchun 130012, China*

Supplementary Table 1. Detailed parameters of the DACs used in the experiments.

Number	Culet size (μm)	Gasket	Composition	Temperature (K)	Pressure (GPa)	Measurement
#Cell-1	80	Re+Al_2O_3/epoxy	La-Ce alloy+ NH_3BH_3	2100	110	SC*, Run-1, XRD
#Cell-2	80	Re+Al_2O_3/epoxy	La-Ce alloy+ NH_3BH_3	2200	97	SC, Run-2
#Cell-3	80	Re+Al_2O_3/epoxy	La-Ce alloy+ NH_3BH_3	2100	85-115	SC, Run-3
#Cell-4	80	Re+Al_2O_3/epoxy	La-Ce alloy+ NH_3BH_3	2300	110	SC, Run-4, XRD, MF**
#Cell-5	80	Re+Al_2O_3/epoxy	La-Ce alloy+ NH_3BH_3	2200	115	SC, Run-5
#Cell-6	60	Re+Al_2O_3/epoxy	La-Ce alloy+ NH_3BH_3	2100	125-172	SC, Run-6
#Cell-S1	80	Re+Al_2O_3/epoxy	La-Ce alloy+ NH_3BH_3	2100	115	XRD

* Superconductivity electrical transport measurement (SC).

** Transport measurements under varying external magnetic fields (MF).

Supplementary Table 2. Calculated and experimental (Cell-4) structures of $P6_3/mmc$-(La,Ce)H_9.

Structure		Lattice Parameters (Å)	Atoms	Atomic coordinates (fractional) x	y	z	Occupancy	Unit cell volume (Å^3)
$P6_3/mmc$-(La,Ce)H_9 (110 GPa)	Calculation	$a = b = 3.721$<br>$c = 5.561$	La(2d)	0.6667	0.3333	0.2500	0.5	66.7
			Ce(2d)	0.6667	0.3333	0.2500	0.5	
			H(12k)	0.1577	0.8424	0.4333	1.0	
			H(4f)	0.3333	0.6667	0.1278	1.0	
			H(2b)	0.0000	0.0000	0.7500	1.0	
	Experiment (Cell-4)	$a = b = 3.762(6)$<br>$c = 5.675(5)$	La(2d)	0.6667	0.3333	0.2500	0.5	69.5(1)
			Ce(2d)	0.6667	0.3333	0.2500	0.5	

Supplementary Table 3. Energy Dispersive X-ray Spectroscopy of La-Ce alloy.

Element	The molar ratio					
	Position 1	Position 2	Position 3	Position 4	Position 5	Position 6
La	50.58%	49.96%	51.32%	51.08%	50.96%	50.74%
Ce	49.42%	50.04%	48.68%	48.92%	49.04%	49.26%
Element	The molar ratio					
	Position 7	Position 8	Position 9	Position 10	Position 11	Position 12
La	50.38%	51.64%	51.93%	49.76%	50.75%	50.30%
Ce	49.62%	48.36%	48.07%	50.24%	49.25%	49.70%

Supplementary Table 4. Inductively Coupled Plasma Atomic Emission Spectroscopy (ICP-AES) working parameters.

Instrument model	ICP-OES:Thermo Fisher iCAP PRO
RF Power/W	1150
Plsama flow/L·min^{-1}	0.5
Auxiliary flow/L·min^{-1}	0.5
Nebulizer flow/L·min^{-1}	12.5
Sample uptake delay/s	30

Supplementary Table 5. Inductively Coupled Plasma Atomic Emission Spectroscopy (ICP-AES) analysis of La-Ce alloy.

Sample	amount (g)	Constant volume (mL)	Element	Solution element concentration (mg/L)	Dilution factor	Digestion solution/ sample solution concentration (mg/L)	Element content (mg/kg)	Element content (%)	The molar ratio (%)
La-Ce alloy	0.0460	25	La	2.718	100	271.800	147717.39	14.772	51.43
			Ce	2.589		258.900	140706.52	14.071	48.57

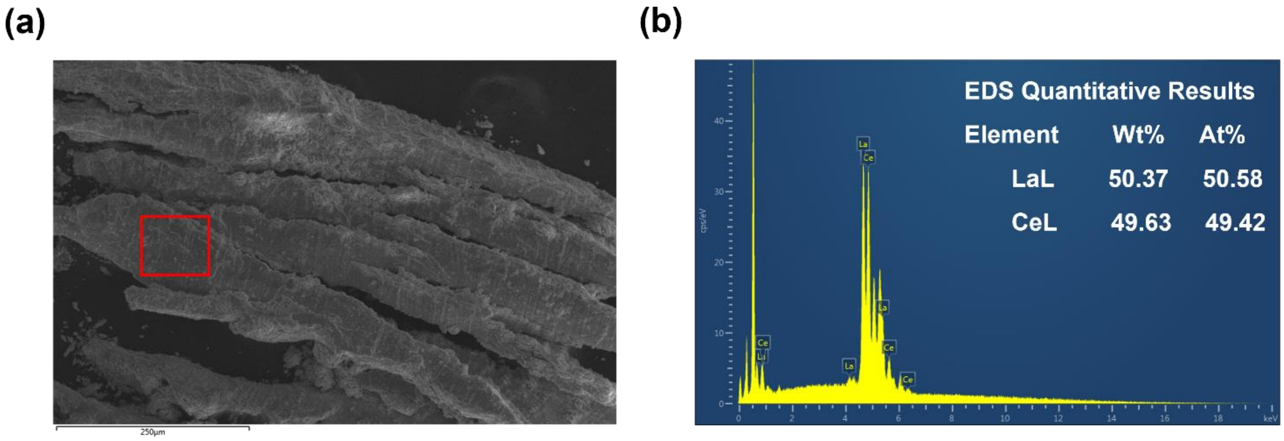

Supplementary Fig. 1. Scanning electron microscopy (SEM) image and energy-dispersive X-ray spectroscopy (EDS) of the La-Ce alloy at position 1. (a) Scanning electron microscope image of La-Ce alloy. **(b)** EDS spectrum from the area highlighted by the red rectangle in the SEM image.

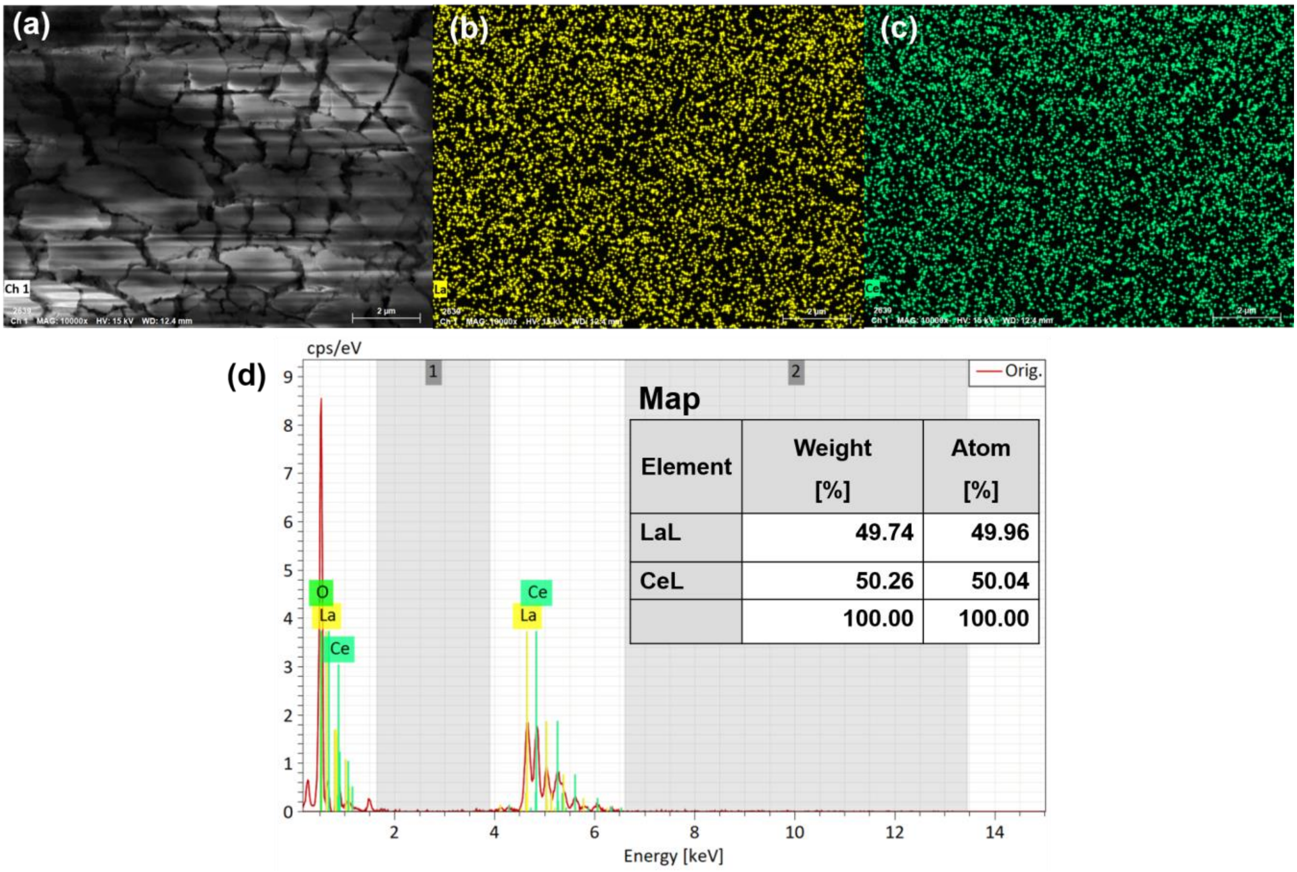

Supplementary Fig. 2. Scanning electron microscopy (SEM) and energy dispersive X-ray spectroscopy (EDS) analysis of the La-Ce alloy in position 2. (a) Scanning electron microscope (SEM) image of La-Ce alloy. **(b)** and **(c)** Element (La and Ce) distribution in (a). **(d)** Components analysis using the EDS in position 2.

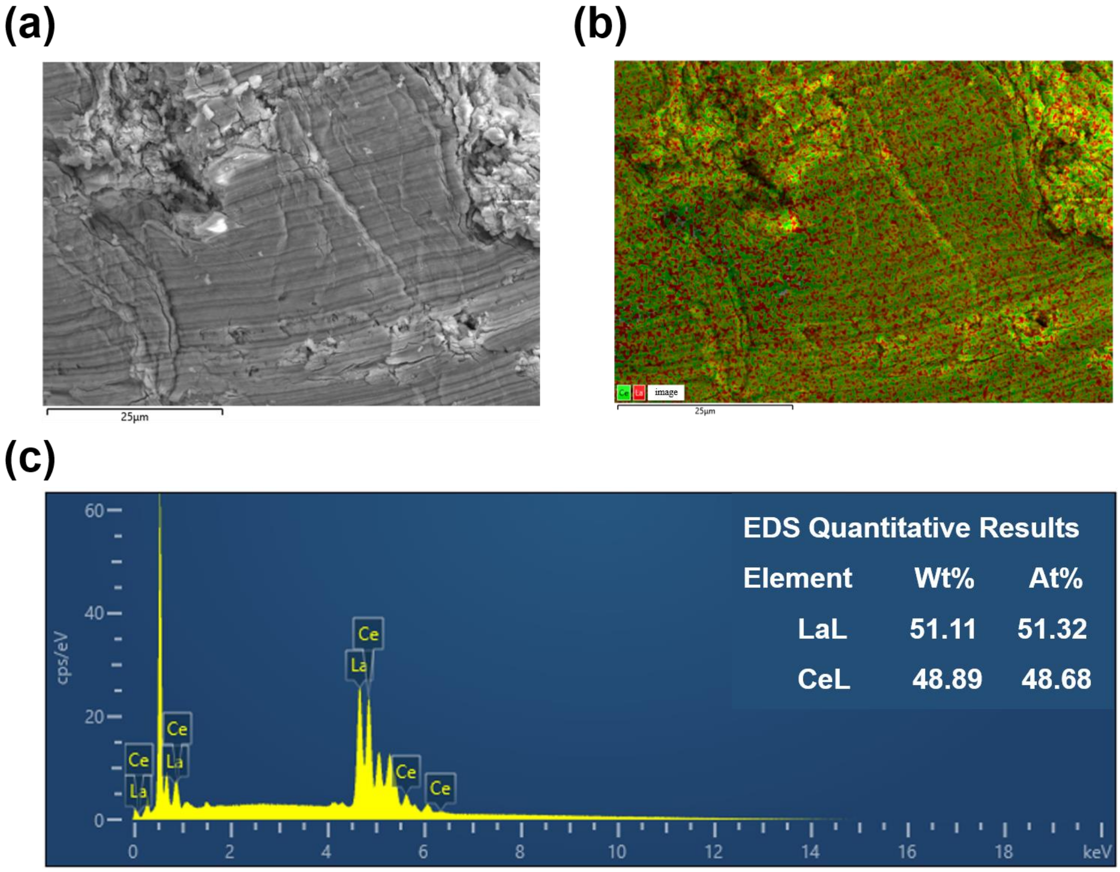

Supplementary Fig. 3. Scanning electron microscopy (SEM) and energy dispersive X-ray spectroscopy (EDS) analysis of the La-Ce alloy in position 3. (a) Scanning electron microscope (SEM) image of La-Ce alloy. **(b)** Element (La and Ce) distribution in (a). **(c)** Components analysis using the EDS in position 3.

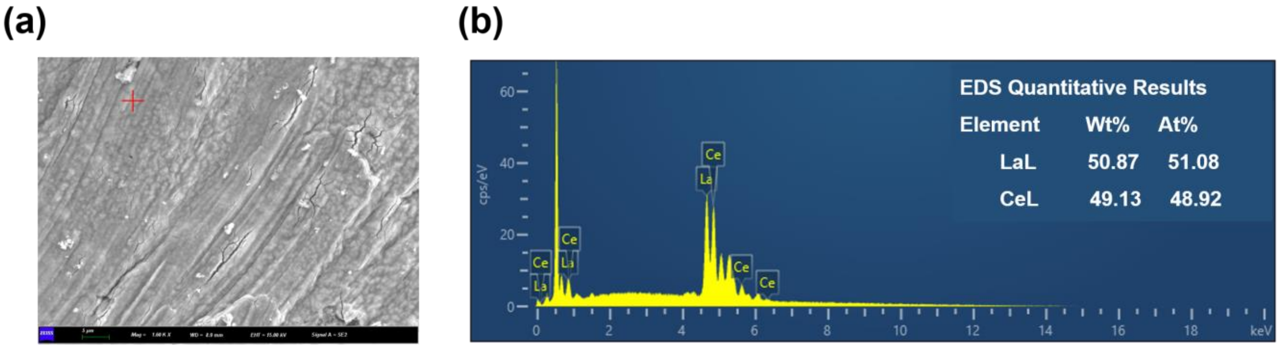

Supplementary Fig. 4. Scanning electron microscopy (SEM) image and energy-dispersive X-ray spectrum (EDS) of the La-Ce alloy at position 4. (a) Scanning electron microscope image of La-Ce alloy. **(b)** EDS spectrum from the point of the red cross in the SEM image.

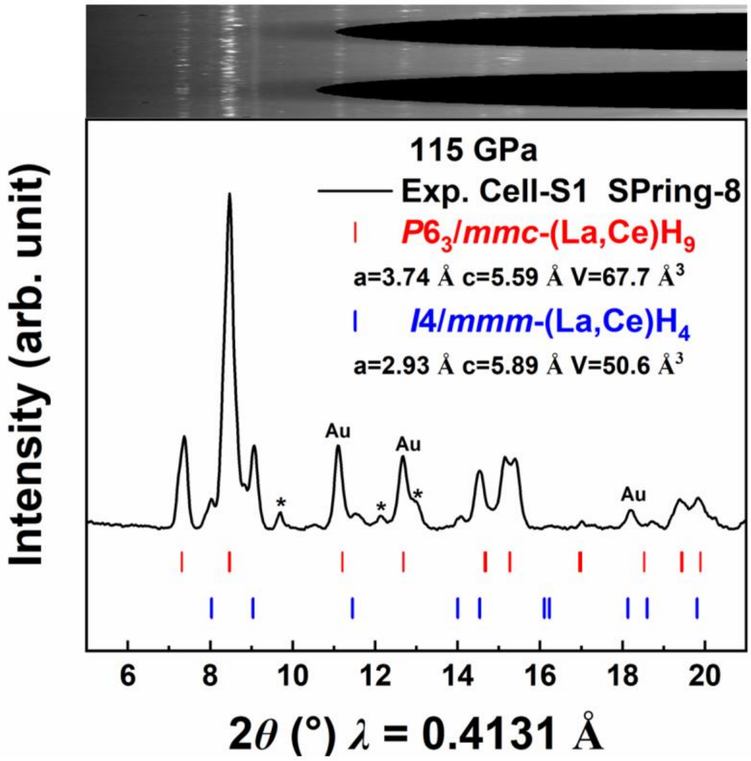

Supplementary Fig. 5. Phase analysis of the composition in Cell-S1 according to synchrotron X-ray diffraction (0.4131 Å) data. The red and blue sticks represent the peak positions of $P6_3/mmc$-(La,Ce)H_9 and $I4/mmm$-(La,Ce)H_4 at 115 GPa, respectively. The weak peak marked with an asterisk may be from undetermined hydride impurity.

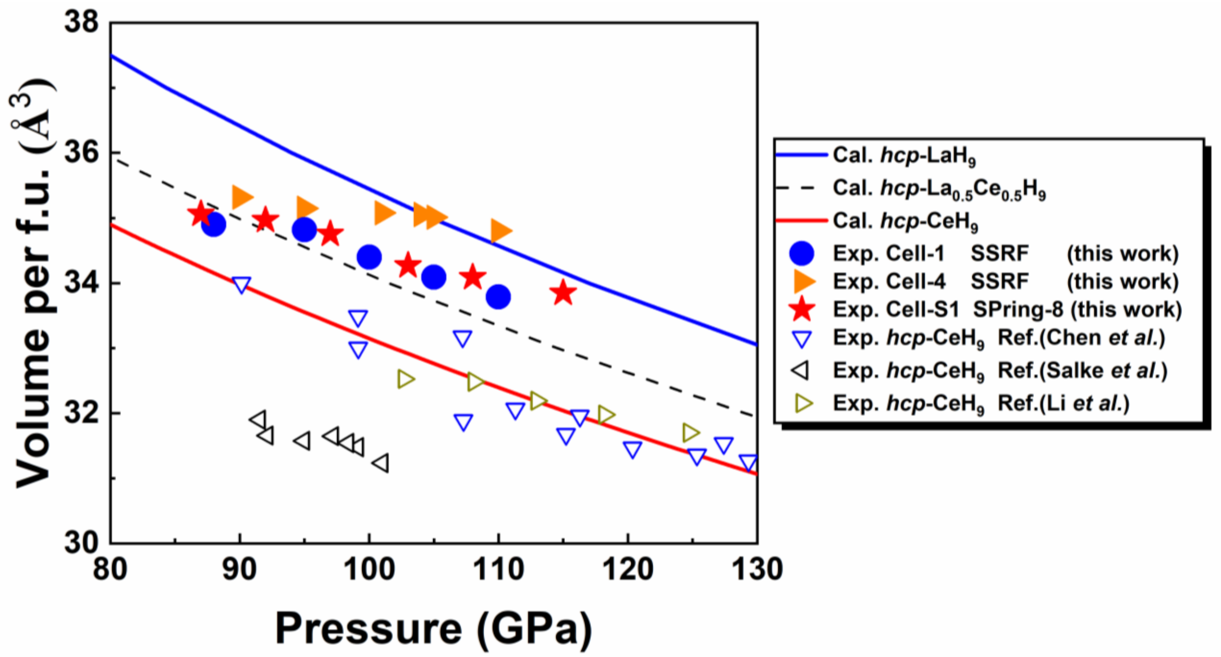

Supplementary Fig. 6. Experimental equation of state (EOS) for different samples and calculated curves of *hcp* LaH_9, $La_{0.5}Ce_{0.5}H_9$, and CeH_9. The experimental results of (La,Ce)H_9 are shown in solid symbols. The experimental volume error may come from the slight deviation of the hydrogen content from the ideal value of

9 for different samples. The hollow triangles are from the Ce-H phases reported by the previous experimental works[1-3]. The *P-V* relationships of these structures are fitted based on the third order Birch-Murnaghan EOS[4]. The hypothetical crystal structures of LaH_9 is isomorphic to the established *hcp* one of CeH_9[5]. One of two Ce atoms in the unit cell of CeH_9 is replaced by La, and the forming ordered $LaCeH_{18}$ ($La_{0.5}Ce_{0.5}H_9$) structure approximately represents the experimental disordered substitutional structure phase. Three (LaH_9, CeH_9, and $La_{0.5}Ce_{0.5}H_9$) structural relaxations were performed using the projector augmented-wave (PAW)[6,7] method with Perdew-Burke-Emzerhof (PBE) exchange correlation function as implanted in the first-principles *Vienna Ab initio Simulation Package*[8] based on density functional theory, where cut-off energy of 700 eV and Brillouin-zone *k*-mesh of $2\pi \times 0.05$ Å^{-1} were used to guarantee energy convergence. The $5s^2 5p^6 5d^1 6s^2$, $5s^2 5p^6 4f^1 5d^1 6s^2$, and $1s^1$ serve as the valence electrons for La, Ce, and H, respectively.

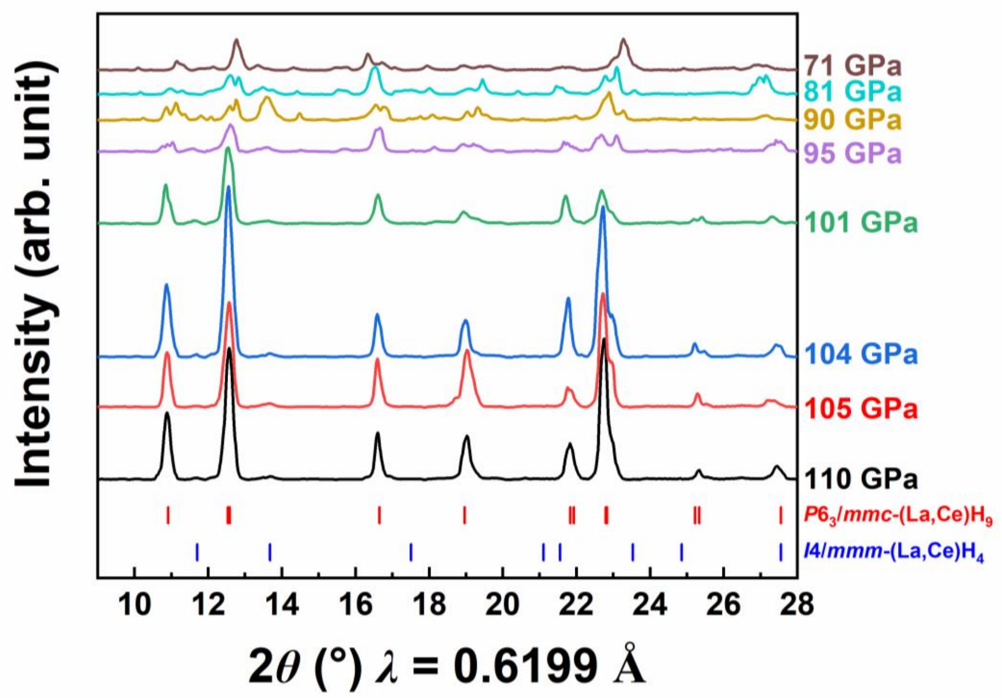

Supplementary Fig. 7. Experimental XRD patterns (Cell-4) during decompression in the pressure range of 71-110 GPa at room temperature. The red and blue sticks represent the peak positions of $P6_3/mmc$-(La,Ce)H_9 and $I4/mmm$-(La,Ce)H_4 at 110 GPa, respectively. The X-ray wavelength is 0.6199 Å.

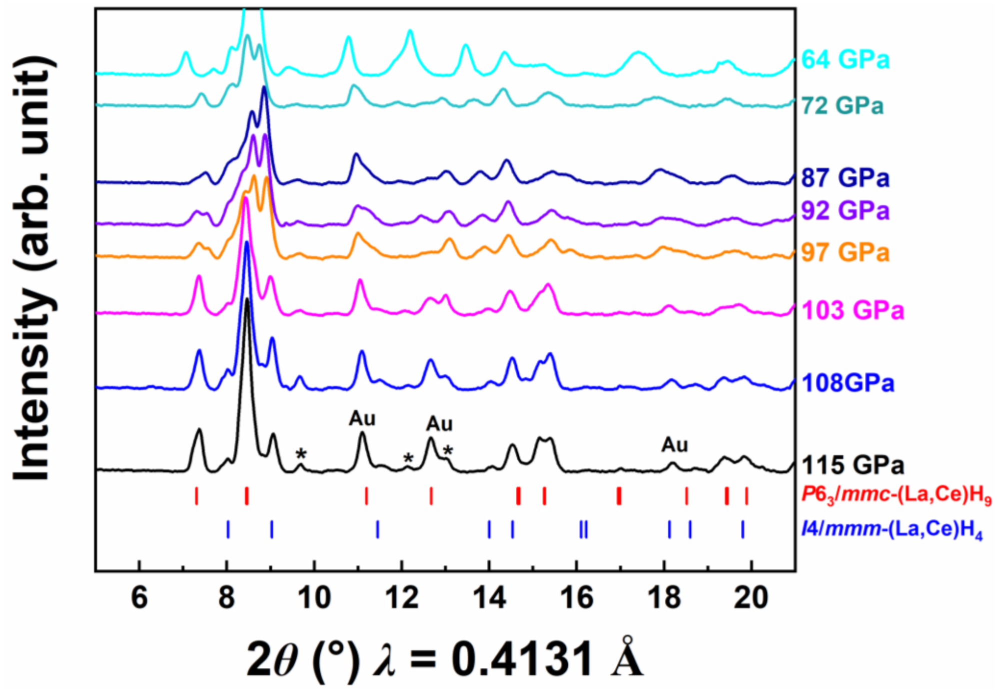

Supplementary Fig. 8. Experimental XRD patterns (Cell-S1) during decompression in the pressure range of 64 - 115 GPa at room temperature. The red and blue sticks represent the peak positions of $P6_3/mmc$-(La,Ce)H_9 and $I4/mmm$-(La,Ce)H_4 at 115 GPa, respectively. The weak peaks marked with asterisks may be from other stoichiometric hydrides. The X-ray wavelength is 0.4131 Å.

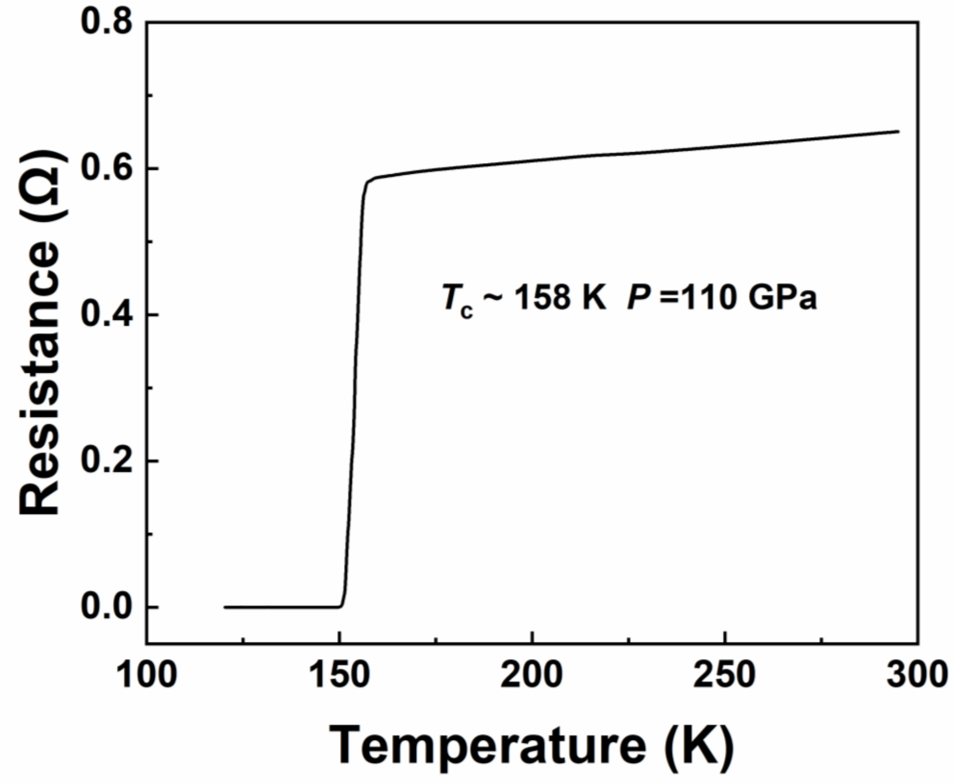

Supplementary Fig. 9. Superconducting transition in warming cycle in Run-1.

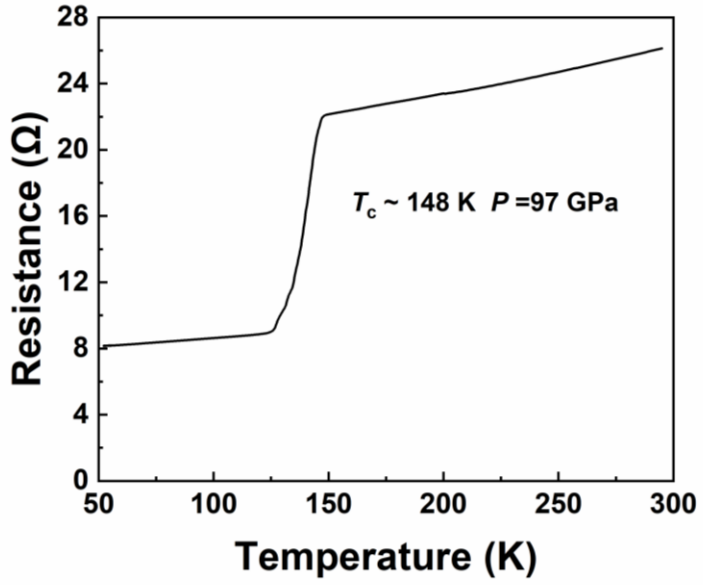

Supplementary Fig. 10. Superconducting transition in warming cycle in Run-2.

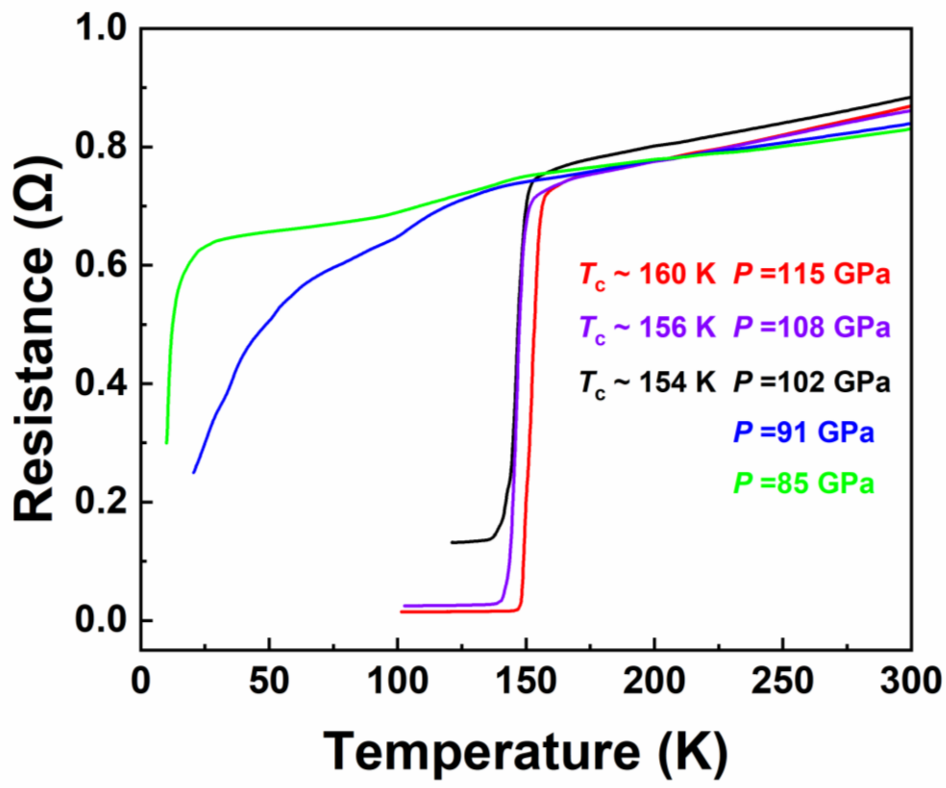

Supplementary Fig. 11. Superconducting transitions in warming cycles in Run-3.

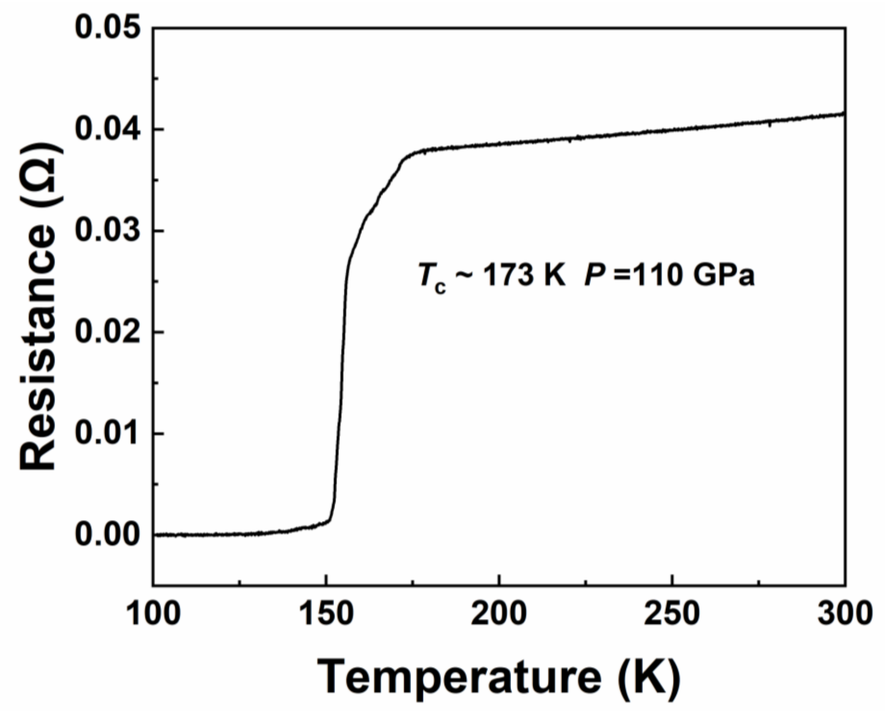

Supplementary Fig. 12. Superconducting transition in warming cycle in Run-4.

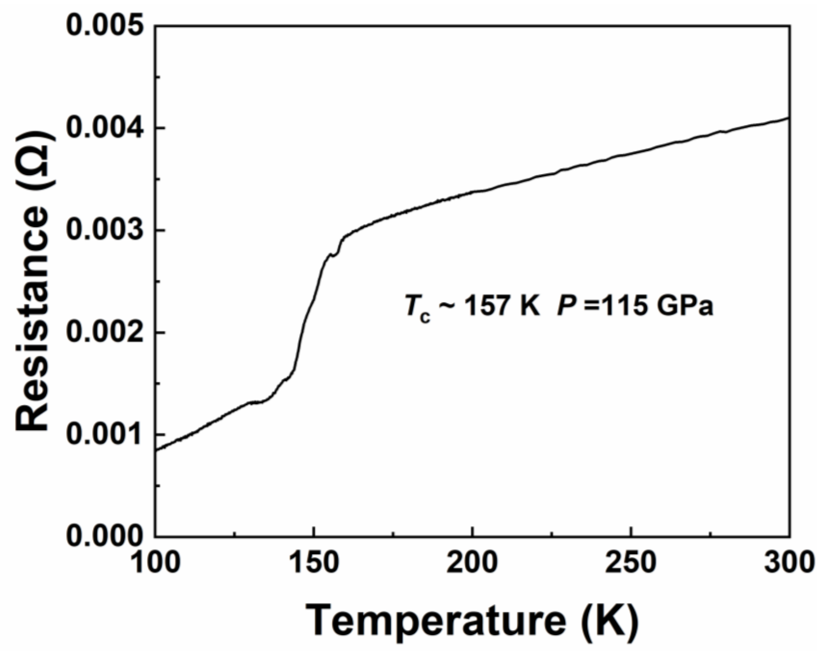

Supplementary Fig. 13. Superconducting transition in warming cycle in Run-5.

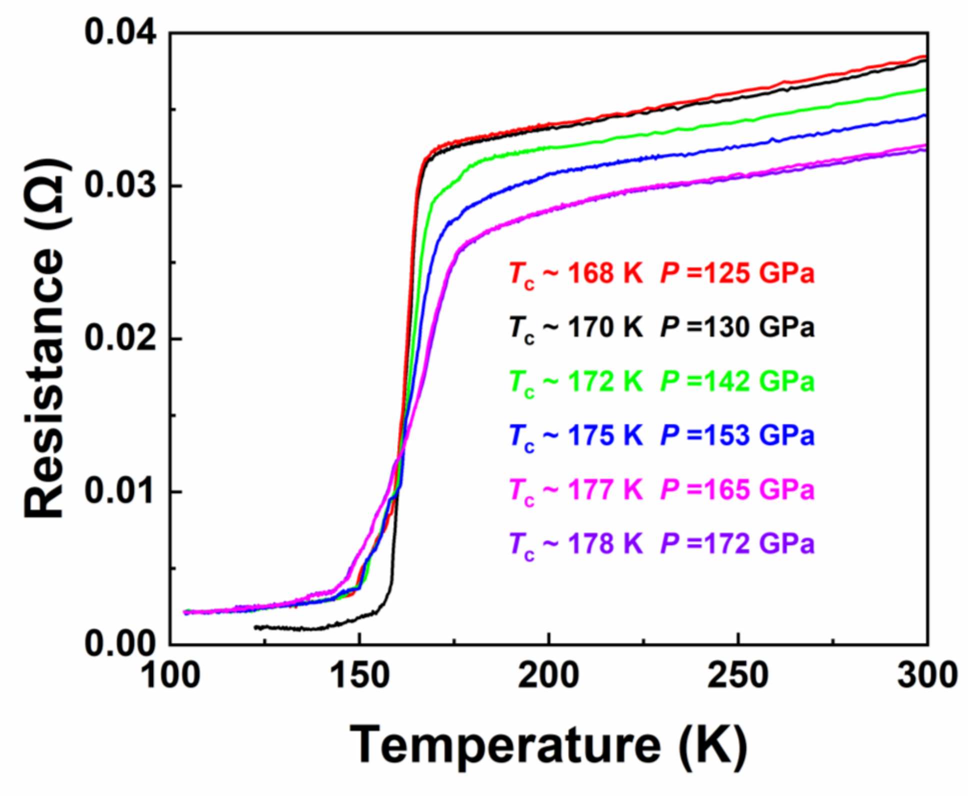

Supplementary Fig. 14. Superconducting transitions in warming cycles in Run-6.

Supplementary References

1. Chen, W. *et al*. High-temperature superconducting phases in cerium superhydride with a T_c up to 115 K below a pressure of 1 megabar. *Phys*. *Rev*. *Lett*. **127**, 117001 (2021).

2. Salke, N. P. *et al*. Synthesis of clathrate cerium superhydride CeH_9 at 80-100 GPa with atomic hydrogen sublattice. *Nat*. *Commun*. **10**, 1-10 (2019).

3. Li, X. *et al*. Polyhydride CeH_9 with an atomic-like hydrogen clathrate structure. *Nat*. *Commun*. **10**, 1-7 (2019).

4. Birch, F. Finite elastic strain of cubic crystals. *Phys*. *Rev*. **71**, 809 (1947).

5. Peng, F. *et al*. Hydrogen clathrate structures in rare earth hydrides at high pressures: possible route to room-temperature superconductivity. *Phys*. *Rev*. *Lett*. **119**, 107001 (2017).

6. Blöchl, P. E. Projector augmented-wave method. *Phys*. *Rev*. *B* **50**, 17953 (1994).

7. Kresse, G. & Joubert, D. From ultrasoft pseudopotentials to the projector augmented-wave method. *Phys*. *Rev*. *B* **59**, 1758 (1999).

8. Kresse, G. & Furthmüller, J. Efficient iterative schemes for *ab initio* total-energy calculations using a plane-wave basis set. *Phys*. *Rev*. *B* **54**, 11169 (1996).
